# Different fetal-neonatal outcomes in siblings born to a mother with Graves-Basedow disease after total thyroidectomy: a case series

**DOI:** 10.1186/1752-1947-4-59

**Published:** 2010-02-19

**Authors:** Antonio Alberto Zuppa, Paola Sindico, Sabrina Perrone, Chiara Carducci, Eleonora Antichi, Giovanni Alighieri, Francesco Cota, Patrizia Papacci, Maria Pia De Carolis, Costantino Romagnoli, Valentina Cardiello

**Affiliations:** 1 Department of Pediatrics, Division of Neonatology, Catholic University of the Sacred Heart, Largo Agostino Gemelli 8, 00168 Rome, Italy

## Abstract

**Introduction:**

We describe three different fetal or neonatal outcomes in the offspring of a mother who had persistent circulating thyrotropin receptor antibodies despite having undergone a total thyroidectomy several years before.

**Case presentation:**

The three different outcomes were an intrauterine death, a mild and transient fetal and neonatal hyperthyroidism and a severe fetal and neonatal hyperthyroidism that required specific therapy.

**Conclusions:**

The three cases are interesting because of the different outcomes, the absence of a direct correlation between thyrotropin receptor antibody levels and clinical signs, and the persistence of thyrotropin receptor antibodies several years after a total thyroidectomy.

## Introduction

Hyperthyroidism occurs in 0.05 to 0.2% of pregnancies. In about 95% of cases it is due to Graves-Basedow disease. In can also be due to Hashimoto's thyroiditis or, less frequently, to toxic adenoma, multinodular toxic goiter, subacute or silent thyroiditis, hydatidiform mole or choriocarcinoma [1-3].

Neonatal hyperthyroidism develops in about 1 to 2% of babies born to mothers suffering from Graves-Basedow disease or, in a few cases, from Hashimoto's thyroiditis [4]. Neonatal hyperthyroidism is usually a transient disorder. It rarely appears at birth, it is more usual within the first week of life. Sometimes it can be lethal because of the development of heart failure [3]. It is usually caused by IgG antibodies stimulating the thyroid stimulating hormone (TSH) receptors of the thyroid gland, which are called thyrotropin receptor antibodies (TRAb). TRAb are able to cross the placental filter and stimulate fetal and neonatal thyroid function [5,6]. These antibodies can persist several years after thyroidectomy [7-9], although, after total surgery, they usually decrease until they finally disappear [9].

We describe three fetal or neonatal outcomes in the offspring of a mother with Graves-Basedow disease. The three cases are interesting because of the different outcomes, the absence of a direct correlation between TRAb levels and clinical signs, and the persistence of TRAb several years after a total thyroidectomy.

## Cases presentation

The mother was a Caucasian Italian woman, diagnosed with Graves-Basedow disease at the age of 14 years. She underwent first subtotal and then total thyroidectomy, and substitutive therapy with L-thyroxine commenced.

Two years later, she was treated with radioiodine therapy because of thyroiditis on thyroid remnants. There was no evidence of thyroid tissue on the following scintigraphic evaluations.

### Case 1

The first pregnancy occurred six years after the total thyroidectomy and four years after the radioiodine therapy. The mother was on substitutive therapy with L-thyroxine (225 μg/day). TRAb levels were not detected during the pregnancy. A Cesarean section was performed at 34 weeks of gestational age (GA), because of intrauterine death of a male fetus. An autopsy was not performed.

### Case 2

**Figure 1:**
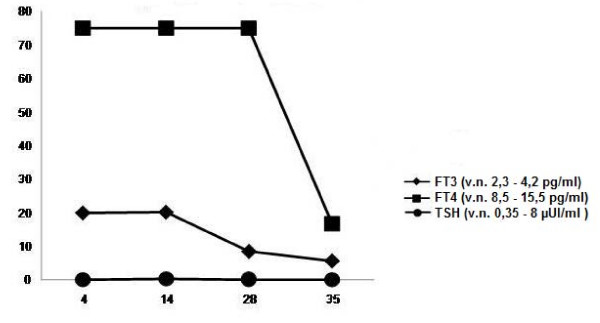
**Serum levels of FT3, FT4 and TSH**.

A year later, the woman became pregnant again. She was still on substitutive therapy with L-thyroxine (225 μg/day) and her hormone levels were within the normal range throughout the whole length of pregnancy. Fetal echocardiographic evaluation was performed one day before the delivery. The report was consistent with mild cardiomegaly and slight sinusal tachycardia, with a fetal heart rate (HR) of 160-170 bpm. TRAb were checked by an enzyme-linked immunosorbent assay (ELISA) with the suspicion of fetal hyperthyroidism. The levels were 32 U/l (normal value [n.v.] <12 U/l). Fetal thyroid ultrasonography was reported to be normal. The following day, the echocardiographic evaluation showed incipient fetal heart failure, severe tricuspid insufficiency, moderate sinusal tachycardia and low amniotic fluid. A Caesarean section was performed at 31 weeks of GA. A female baby was born with an Apgar score of 8-9 and a birth weight of 1870 g. She was transferred to the neonatal intensive care unit. On her 1st day of life (DOL), TRAb were 24 U/l (n.v. <12 U/l). Thyroid hormones and TSH levels (Figure 1) were consistent with neonatal hyperthyroidism (fT3 19.9 pg/ml (n.v. 2.3-4.2), fT4 >75 pg/ml (n.v. 8.5-15.5), TSH 0.03 UI/ml (n.v. 0.35-8)). The baby developed the following clinical signs of hyperthyroidism: considerable weight loss (-12% compared with birth weight), inconsolable crying, irritability and tachycardia at rest (HR 180-190 bpm). Echocardiogram was normal and was not in agreement with prenatal data. Thyroid ultrasonography results were within the normal range. Both clinical signs and thyroid hormone levels normalized during hospitalization and therapy was not required. The baby was discharged on the 36^th ^DOL. TRAb levels were 2 U/l (n.v. <1.5 U/l).

### Case 3

The third pregnancy occurred nine years after total thyroidectomy and seven years after radioiodine therapy. The mother was receiving substitutive therapy with L-thyroxine (225 μg/day). Hormone and TSH levels were within the normal range throughout the whole pregnancy. Lugol's solution (potassium iodine) at the dosage of 8 mg/day was administered to the mother, starting in the 25th week of GA and continuing for 20 days, because of fetal tachycardia. From the 31st week until delivery, methimazole (20 mg/day) was added because of persistent fetal tachycardia. Lugol's solution (8 mg/day) was added during the last two weeks. TRAb levels, checked with a radio immunosorbent test (RIA), were about 400 UI/l at 19 and 29 weeks of GA, respectively (n.v. <10 UI/l). Fetal thyroid ultrasonography and echocardiography were normal. At 33 weeks of GA, a female baby was born by Caesarean section, which was carried out due to the persistent fetal tachycardia. The birth weight was 2200 g and the Apgar score was 8-9. Echocardiographic evaluation at birth showed a patent ductus arteriosus with initial overload of left cardiac sections and slight tricuspid insufficiency. All these findings disappeared on the 6th DOL. HR was 160-180 bpm. Thyroid hormones levels were within the normal range and TRAb levels were 35 U/l (n.v. <12 U/l).

A considerable weight loss was detected (-10.5% compared with birth weight).

**Figure 2:**
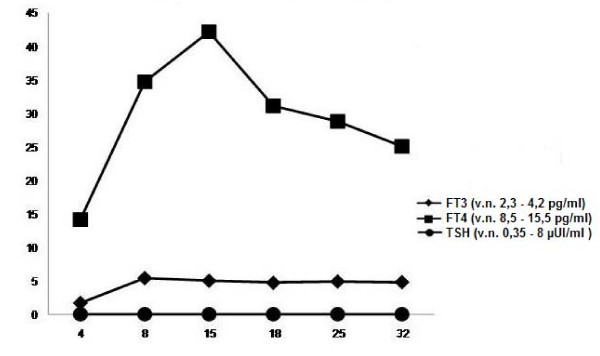
**Serum levels of FT3, FT4 and TSH**.

By the 7th DOL, the baby was extremely irritable with inconsolable crying. At that point, thyroid hormones and TSH levels (Figure 2) were consistent with hyperthyroidism (fT3 5.4 pg/ml (n.v. 2.3-4.2), fT4 34.7 pg/ml (n.v. 8.5-15.5), TSH 0.03 UI/ml (n.v. 0.35-8)), probably due to maternal antithyroid drug clearance. Lugol's solution was started (8 mg/3 times a day). On the 9th DOL, the newborn presented supraventricular parossistic tachycardia (HR 330 bpm). Diving reflex was necessary to reduce HR to 180 bpm. The dosage of Lugol's solution was increased to 24 mg/3 times a day and oral administration of diazepam was necessary, because of a persistent clinical pattern of hyperthyroidism (tachycardia, supraventricular extrasystoles, hyperexcitability, irritability, inconsolable crying and vomiting). On the 13th DOL, propranolol was started (1 mg/kg/day, in 4 daily doses), due to persistent tachycardia. It was withdrawn on the 28th DOL. The baby was discharged at one month old and discontinued Lugol's solution after a week. TRAb levels were not dosable at that point. Thyroid hormones levels were normal (fT3 3.3 pg/ml (n.v. 2.3-4.2), fT4 11 pg/ml (n.v. 8.5-15.5)) at two months of life. Thyroid ultrasonography consistently showed normal results.

## Discussion

**Newborns **of mothers with autoimmune thyroid diseases, especially Graves-Basedow disease and Hashimoto thyroiditis, are at risk of developing thyroid dysfunction. Fetal hyperthyroidism may cause intrauterine growth restriction, intrauterine death, preterm birth, fetal tachycardia and non immune hydrops [10].

Neonatal clinical signs of hyperthyroidism include: goitre, irritability, periorbital oedema, exophthalmos, craniosynostosis, microcephaly, tachycardia, arrhythmias, cardiac failure, voracious appetite, weight loss, diarrhoea, vomiting, sweating, flushing, hepatosplenomegaly, lymphadenopathy, thrombocytopenia and hyperviscosity [8].

In our three cases, we report various clinical presentations, from fetal death to neonatal hyperthyroidism with different grade of severity.

In the first case, the pregnancy was not optimally monitored, so fetal death could be a consequence of unknown and untreated fetal hyperthyroidism due to TRAb transplacental passage. It is likely that the circulating TRAb were already present, because they were detected in the subsequent pregnancies and just one year later.

The second pregnancy was well monitored. The mother was treated with L-thyroxine, which ensured a normal thyroid function. The newborn developed signs of a mild neonatal hyperthyroidism (sinusal tachycardia, abnormal thyroid hormones and TSH levels, considerable weight loss, irritability) but they were transient and solved without any therapy.

In the third case, the TRAb of the mother, reported to be at normal levels, seemed to be higher than during the second pregnancy, although the values were not comparable because the different evaluation methods. However, the TRAb levels at birth were similar in the two siblings assayed with the same method.

This suggests that there is not a close correlation between TRAb levels and fetal and/or neonatal clinical features, which indicates that all newborns with TRAb, regardless to their value, should be monitored carefully. After a total thyroidectomy, TRAb levels should decrease, because of the lack of antigen stimulation; one mechanism of TRAb persistence could be microchimerism.

During pregnancy, fetal antigens could pass through the placental filter and become triggers for TRAb production [11]. Pregnancy is the most common source of microchimerism. Fetal cells or DNA can persist in women for several years after delivery [12]. Fetal microchimerism could contribute to pathogenesis of autoimmune diseases [13]. In our patient, the first pregnancy (intrauterine death) could have caused the passage of fetal cells and/or antigenic fragments able to induce and maintain TRAb production, even after the total thyroidectomy and the radioiodine therapy. The same mechanism could have occurred between second and third pregnancy.

We would have expected a better neonatal outcome in the third pregnancy because the decrease of TRAb levels. Instead we observed worsening of clinical fetal and neonatal manifestations and an increase of TRAb levels, which were reported to be in the normal range.

## Conclusion

Our experience demonstrates that it is difficult to foresee a close temporal correlation between maternal thyroidectomy and fetal and/or neonatal outcome. It is important to consider occurrence of fetal and neonatal hyperthyroidism even several years after a total thyroidectomy. Surveillance of both mother and fetus and/or neonate using a multidisciplinary approach is mandatory.

## Abbreviations

DOL: day of life; GA: gestational age; HR: heart rate; n.v: normal value; TRAb: thyrotropin receptor antibodies; TSH: thyroid stimulating hormone.

## Consent

Written informed consent was obtained from the patient for publication of these case reports and accompanying images. A copy of the written consent is available for review by the journal's Editor-in-Chief.

## Competing interests

The authors declare that they have no competing interests.

## Authors' contributions

AAZ designed and carried out the research, and was a major contributor in writing the manuscript. PS analyzed the patient data, carried out the research and wrote the manuscript. SP carried out the research and wrote the manuscript. CC, EA and GA carried out the research; FC analyzed and interpreted patient data; PP carried out the research, and analyzed and interpreted patient data. MPDC carried out the research and wrote the manuscript; CR designed the research. VC carried out the research and wrote the manuscript. All authors read and approved the final manuscript.
